# Plasticity of Lateral Root Branching in Maize

**DOI:** 10.3389/fpls.2019.00363

**Published:** 2019-03-29

**Authors:** Peng Yu, Frank Hochholdinger, Chunjian Li

**Affiliations:** ^1^ Crop Functional Genomics, Institute of Crop Science and Resource Conservation (INRES), University of Bonn, Bonn, Germany; ^2^ Department of Plant Nutrition, College of Resources and Environmental Science, China Agricultural University, Beijing, China

**Keywords:** maize, lateral root, plasticity, nitrate, water

## Abstract

Extensively branched root systems can efficiently capture soil resources by increasing their absorbing surface in soil. Lateral roots are the roots formed from pericycle cells of other roots that can be of any type. As a consequence, lateral roots provide a higher surface to volume ratio and are important for water and nutrients acquisition. Discoveries from recent studies have started to shed light on how plant root systems respond to environmental changes in order to improve capture of soil resources. In this Mini Review, we will mainly focus on the spatial distribution of lateral roots of maize and their developmental plasticity in response to the availability of water and nutrients.

## Introduction

Maize forms a structurally and functionally complex root system composed of different root types (Hochholdinger et al., 2017) to efficiently acquire water and nutrients (Lynch, 2013) and to tolerate biotic and abiotic stresses (Lynch et al., 2014). Lateral roots of different orders are the most eminent root type for nutrient and water uptake from soil because of their high surface to volume ratio (Rogers and Benfey, 2015). Compared to other root types, lateral roots display the highest developmental plasticity when exposed to unfavorable environmental conditions (Yu et al., 2014a, 2016a). The formation and spatial distribution of lateral roots, e.g., lateral root branching is the most important factor governing root system architecture and soil exploration in plants (Atkinson et al., 2014). Thus, genotypes with lateral root defects display a strong inhibition of nutrient uptake and biomass production in crops (Yu et al., 2016a). The molecular mechanisms and hormonal crosstalk involved in lateral root formation and positioning has been extensively studied in the model plant *Arabidopsis thaliana* (Möller et al., 2017; Ötvös and Benková, 2017). Genetic and molecular control of lateral root initiation and formation in maize has been summarized in the recent review (Yu et al., 2016a). In this Mini Review, we provide an update on the molecular mechanisms involved in the lateral root branching response to environmental cues such as nutrients and water in maize.

## Architectural Responses of Lateral Roots to the Availability of Soil Resources

The significantly higher surface area of the lateral roots compared to their parental roots is a major determinant that is instrumental for water uptake in maize (Ahmed et al., 2016). Lateral roots are efficient in the short-distance exploitation and transport of water from soil to the vasculature in young and adult maize plants (Ahmed et al., 2016, 2018). Genotypic differences in lateral root branching and their vertical distribution along the root system are a measure for drought tolerance in soil (Hund et al., 2009). Maize genotypes with reduced lateral root branching have been shown to be highly tolerant against drought under both greenhouse and field conditions (Zhan et al., 2015). A possible explanation for this observation is the negative correlation between lateral root branching and axial root elongation (Lynch, 2015). In maize, distinct orders of lateral roots make up the majority of total length of the root system (Lynch, 2013). Different plant species display distinct responses to nitrogen and phosphorus starvation with respect to their lateral root branching patterns, although dicot and monocot plants show similar patterns of lateral root spacing along the primary root under optimal conditions (Chen et al., 2018). The optimal branching density of lateral roots in maize has been predicted by the functional-structural model *SimRoot* based on the observation that nutrient acquisition is proportional to the spatial availability and mobility of resources in the soil profile (Postma et al., 2014). Genotypes with sparsely distributed and long lateral roots are optimal for nitrate acquisition, whereas genotypes with densely spaced and short lateral roots are optimal for phosphorus acquisition in maize (Postma et al., 2014). Recent results in maize have indicated that genotypes with higher lateral root branching density display significantly increased phosphorus acquisition under phosphorus-deficient conditions (Liu et al., 2013; Jia et al., 2018). By contrast, maize genotypes with few and long lateral roots are more competent for nitrogen uptake than genotypes with many and short lateral roots under suboptimal nitrogen concentrations in soil (Zhan and Lynch, 2015). Thus, availability of soil nutrients and water determines compensatory growth and patterning of lateral roots along the parental root axes.

## Lateral Root Branching in Response to Patchy Soil Resources

**Figure 1:**
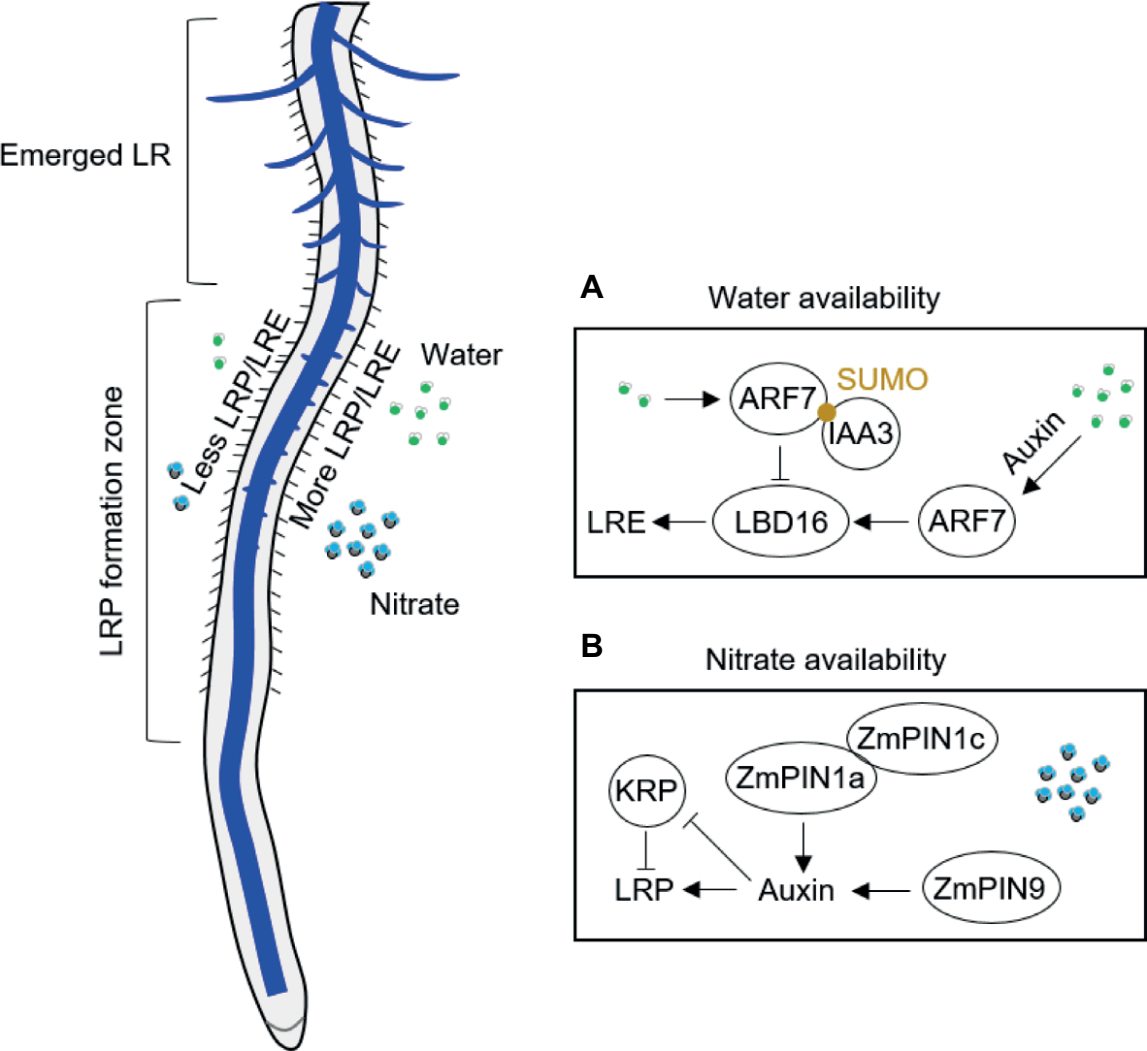
Schematic illustration of lateral root branching response to availability of water in *A. thaliana* **(A)** and of nitrate in maize **(B)**. ARF, auxin response factor; IAA3, Aux/IAA3 (Aux/indole-3-acetic acid protein 3); KRP, Kip-related protein; LBD, LATERAL ORGAN BOUNDARIES (LOB) domain; LR, lateral root; LRE, lateral root emergence; LRP, lateral root primordia; PIN, PIN-Formed; SUMO, small ubiquitin-like modifier.

Lateral root branching patterns reflect the uneven distribution of water and nutrients in soil (Robinson, 1994). Plants adapt to heterogeneous water conditions by altering their lateral root branching in contact with water by using “hydropatterning” response (Bao et al., 2014; Robbins and Dinneny, 2018). High-resolution non-invasive microcomputed tomography imaging has revealed that the formation and patterning of lateral roots is highly responsive to local water availability in *A. thaliana* and crop species (Bao et al., 2014; Robbins and Dinneny, 2015; Orman-Ligeza et al., 2018). Xerobranching, describing the repression of lateral root branching when root tips are not in contact with wet soil, suggests that abscisic acid is involved as key signal regulating branches of lateral roots in their local microenvironment (Orman-Ligeza et al., 2018). For hydropatterning, high availability of water results in the induction of auxin biosynthesis and transport, independent of endogenous abscisic acid signals (Bao et al., 2014; Figure 1A). A recent study with *A. thaliana* demonstrated that hydropatterning is dependent on SUMO-mediated posttranslational regulation of auxin signaling pathway (ARF7/IAA3) controlling lateral root branching pattern in response to water availability (Orosa-Puente et al., 2018; Figure 1A). ARF7 transcription factor induces asymmetric expression of its target gene LATERAL ORGAN BOUNDARIES (LOB) domain 16 (LBD16) in lateral root founder cells (Orosa-Puente et al., 2018; Figure 1A). Future work will be necessary to understand how the water and nutrient signals are integrated to regulate lateral root branching in response to local water/nutrients availability (Giehl and von Wirén, 2018). Through a combination of empirical and mathematical-modeling approaches in maize, it has been shown a central role of tissue growth and developmental competence, which is necessary to sustain the normal hydropatterning, although the molecular mechanism is unknown in crop plants (Robbins and Dinneny, 2018). This result implies that the water requirement for fast developing tissue is an important contribution process on water perception and developmental reprogramming during the postembryonic root development.

Lateral root formation in response to systemic and local nitrate signaling has been recently summarized in *A. thaliana* (Sun et al., 2017). A series of experiments have demonstrated that local nitrate supply can considerably stimulate lateral root production also in crops, such as maize (Wang et al., 2004; Guo et al., 2005; Liu et al., 2008, 2010), rice (Huang et al., 2015), barley (Drew et al., 1973), and wheat (Hackett, 1972). Hydroponics allows local nutrient application under well-controlled conditions, which can also avoid influences of other factors such as physical properties of soil and microbes. Local application of nutrients in hydroponics includes two strategies: one is the vertical split system that the middle part of the root was supplied with high nutrients along the longitudinal axis (Drew et al., 1973); another strategy is horizontal split system that the axial roots are separated into different compartments or rhizoslides with diverse nutrient levels (Yu et al., 2014b; Dina in’t Zandt et al., 2015). The complex nature of the root system of maize plants makes split-root experiments challenging. Most common ways to carry out such experiments is by placing the primary root into one compartment and the other root types are placed into the other one (Yu et al., 2014b, 2015, 2016b) or the crown roots are equally separated and cultured in different compartments after removing the primary and seminal roots (Wang et al., 2004; Guo et al., 2005; Liu et al., 2008, 2010). Vertical hydroponics experiments by Drew and his colleagues demonstrated that lateral root formation in barley depends on the dose and type of nutrient application (reviewed in Yu et al., 2014a). Both length and density of lateral roots are significantly induced by local high nitrate (control: 0.01 mM; high nitrate 1 mM) in barley seedlings (Drew et al., 1973). By contrast, only elongation of the lateral roots on primary root is induced in maize seedlings split supplied by local high nitrate (control: 0.5 mM; high nitrate 4 mM) in 7-day-old maize seedlings grown in the left-right hydroponic system (Yu et al., 2014b). This is consistent with the lateral root formation from the crown roots of maize, that localized nitrate mainly induced lateral root formation but little effects on the density of lateral roots in both hydroponics (Liu et al., 2010) and rhizoslides (Dina in’t Zandt et al., 2015). This is further validated by a microarray analysis of pericycle cells indicating common mechanisms for lateral root initiation in maize primary and crown roots (Jansen et al., 2013). Different responses on lateral root branching between barley and maize can be explained by species-specific responses to nitrate but also by different developmental stages of root types surveyed in these plant species. Thus, lateral root specific responses to local nitrate depend on the developmental stage when certain root type is formed. For instance, shoot-borne roots specifically initiated during silking form more lateral roots in response to localized nitrate supply than other root types including the shoot-borne roots formed before silking (Yu et al., 2015, 2016b). This divergent finding can be explained by the higher shoot demand for nutrients during and after silking (Yu et al., 2014b) but also the possibility of specific hormone signaling from the reproductive process (Yu et al., 2016b). For example, basipetal auxin transport is facilitated by ZmPIN1a and ZmPIN1c in response to local nitrate supply (Yu et al., 2016b; Figure 1B). Moreover, monocot-specific *PIN9* gene in phloem pole cells of shoot-borne roots at silking modulates auxin efflux to pericycle cells and subsequent cell cycle activation by alleviating the inhibition of Kip-related proteins (KRP) coding genes in maize (Yu et al., 2016b; Figure 1B). Moreover, CPP-like (cysteine-rich polycomb-like) transcription factors have been found specifically enriched in brace roots of maize, which may play an important role in development of reproductive organs and control of cell division in plants (Yu et al., 2016b). It would be interesting to compare the responses of different root types to local nitrogen supply at the flowering stage in order to answer whether in maize this divergent response is root type specific and/or developmental stage dependent.

A study with *A. thaliana* mutants with reduced number of lateral roots indicate that complex architecture and branching pattern of lateral roots are mainly required for the acquisition of immobile resources, such as phosphate, whereas mobile ions like nitrate can be effectively taken up even by restricted root systems (Fitter et al., 2002). This raises the hypothesis that root proliferation in nutrient-rich patches could be more important for the enhanced capture of immobile than mobile ions. In fact, field studies have suggested that enhanced root proliferation in nutrient-rich patches of ammonium and phosphate during seedling stage and adult development is essential for improving phosphate uptake and ultimately grain yield (Jing et al., 2010; Li et al., 2012, 2016; Ma et al., 2013). One possible explanation for this observation could be indirect effects of solubilization of mineral phosphate by rhizosphere acidification using ammonium fertilization. Alternatively, fine lateral root proliferation substantially increases exudate secretion to the rhizosphere, which can be used as the carbon source for beneficial interactions of the microbiome at the root-soil interface.

## Root Type-Specific Branching Patterns

Recent experiments highlight root type-specific transcriptomic, anatomical, and physiological differences in maize (Tai et al., 2015; Ahmed et al., 2018). Distinct root types of maize show diverse branching responses to nitrate and also host different fungal taxa in their axial and lateral roots (Yu et al., 2016b, 2018). A novel phenotyping approach demonstrates distinct growth rates of three types of lateral roots contribution to the random patterning of lateral root formation in pearl millet and maize (Passot et al., 2018). This phenomenon raises the question whether lateral root types with divergent lengths show distinct competences of their corresponding pericycle cells for dividing. To further study this question, diverse inbred lines with natural variation for lateral root patterning and spacing could be studied in maize.

## Conclusions and Perspectives

Lateral root branching of maize plants grown in soil is root type specific and depends on hormonal crosstalk and signal transduction based on local sensing of water and nutrients. Nevertheless, these molecular processes have not been understood in full detail at the cellular level. Therefore, systemic cell-type-specific analyses in different root types will be instrumental to clarify the identity of pericycle and endodermis cells in maize in response to local water and nutrient supply (Kortz et al., 2019). In particular, root type has to be considered when studying the mechanism of lateral root formation as maize has a unique architectural pattern in comparison to the other crop species (Burton et al., 2013; Tai et al., 2015; Yu et al., 2016a). Signal transduction induced by water and nutrients at the root-soil interface needs to be explored in large genetic populations on the molecular level and by advanced *in situ* imaging (van Dusschoten et al., 2016; Tardieu et al., 2017). To this end, understanding the reprogramming of lateral root formation and architectural plasticity in response to water and nutrient availability in the context of yield acquisition and resource use efficiency can be relevant for rational breeding approaches.

## Author Contributions

PY, FH and CL contributed to the writing of this minireview.

### Conflict of Interest Statement

The authors declare that the research was conducted in the absence of any commercial or financial relationships that could be construed as a potential conflict of interest.
